# Public beliefs about the consequences of living with obesity in the Republic of Ireland and Northern Ireland

**DOI:** 10.1186/s12889-022-14280-9

**Published:** 2022-10-13

**Authors:** Eleni Spyreli, L McGowan, E Heery, A Kelly, H Croker, C Lawlor, R O’Neill, CC Kelleher, M McCarthy, P Wall, MM Heinen

**Affiliations:** 1 Centre for Public Health, School of Medicine, Dentistry and Biomedical Science, Queen’s University Belfast, Belfast, UK; 2 Library and Research Service, Oireachtas, Houses of the Oireachtas Service, Dublin, Ireland; 3 Population, Policy and Practice Research and Teaching Department, UCL Great Ormond Street Institute of Child Health, University College London, London, UK; 4 National Nutrition Surveillance Centre, University College Dublin, Dublin, Ireland; 5 Cork University Business School, University College Cork, Cork, Ireland

**Keywords:** Living with overweight and obesity, Obesity beliefs, Island of Ireland

## Abstract

**Background:**

This study aimed to capture public beliefs about living with obesity, examine how these beliefs have changed over time and to explore whether certain characteristics were associated with them in a nationally representative sample of adults from the Republic of Ireland (RoI) and Northern Ireland (NI).

**Methods:**

A cross-sectional survey employed a random quota sampling approach to recruit a nationally representative sample of 1046 adults across NI and RoI. Telephone interviews captured information on demographics; health behaviours & attitudes; and beliefs about the consequences of obesity (measured using the Obesity Beliefs Scale). Univariable analyses compared beliefs about the consequences of living with obesity between participants with a self-reported healthy weight and those living with overweight or obesity, and non-responders (those for whom weight status could not be ascertained due to missing data). Multiple linear regression examined associations between obesity-related beliefs and socio-demographics, self-rated health and perceived ability to change health behaviours. Multiple linear regression also compared changes in obesity-related beliefs between 2013 and 2020 in the RoI.

**Results:**

Higher endorsement of the negative outcomes of obesity was significantly associated with living with a healthy weight, higher self-rated health, dietary quality and perceived ability to improve diet and physical activity. Those who lived with overweight, with obesity and non-responders were less likely to endorse the negative consequences of obesity. Those living with obesity and non-responders were also more likely to support there is an increased cost and effort in maintaining a healthy weight. Comparison with survey data from 2013 showed that currently, there is a greater endorsement of the health benefits of maintaining a healthy weight (*p* < 0001), but also of the increased costs associated with it (*p* < 0001).

**Conclusion:**

Beliefs about the consequences of maintaining a healthy body weight are associated with individuals’ weight, self-rated health, diet and perceived ease of adoption of dietary and exercise-related improvements. Beliefs about the health risks of obesity and perceived greater costs associated with maintaining a healthy weight appear to have strengthened over time. Present findings are pertinent to researchers and policy makers involved in the design and framing of interventions to address obesity.

**Supplementary information:**

The online version contains supplementary material available at 10.1186/s12889-022-14280-9.

## Background

Increasing rates of overweight and obesity pose a complex challenge for public health and the economy. There is mounting evidence that individuals with excess body weight have an increased risk of morbidity and mortality [1]. Indicatively, obesity has been associated with 2- to 4-year reduction in life expectancy [2, 3] and up until the beginning of year 2020, obesity has been responsible for up to 13% of total deaths in Europe [4].

Overweight and obesity also have social implications. Levels of weight (obesity) bias (a term referring to negative attitudes/beliefs about a person based on their body size resulting in social stereotypes and misconceptions about obesity) have increased alongside the rise in obesity levels [5]. This can lead to actions against people with obesity, such as exclusion, marginalisation and inequalities (in healthcare, education, workplace, etc.) [6]. The negative consequences of experiencing stigmatisation due to one’s body weight are wide-ranging, and may include internalising negative beliefs about oneself due to body size, engaging in unhealthy eating behaviours (promoting further weight gain) as a coping response, and disengagement with healthcare services [7]. Growing evidence also links obesity stigma to psychological stress, which in turn can lead to impairment of physical and psychological health [8, 9].

Even though the nature of obesity is multifactorial, with genetic predisposition, social, environmental and psychological aspects playing a contributing role in its development [10, 11], promoting behavioural modification through diet and physical activity has shown some success in improving health outcomes and managing weight [12–16]. Adopting certain health behaviours, such as dietary and physical activity-related ones, can be influenced, in part, by an individual’s beliefs about the negative and positive outcomes of these behaviours [17]. In this way, individuals who believe that harm could result from engaging in behaviours that can lead to an increased body weight (e.g. regularly consuming excess calories from foods high in fat and sugar) may be less likely to adopt such behaviours.

National obesity policies in the Republic of Ireland and Northern Ireland aim to address obesity at a population level by changing public views associated with living with excess weight and healthy weight (i.e. by illustrating the potentially negative health consequences from living with excess weight, or highlighting the benefits of a healthy diet), to stimulate engagement in health-promoting behaviours [18, 19]. A survey conducted in 2013 in the Republic of Ireland demonstrated that most of these policies (such as education on healthy eating in schools, food labels with calorie and nutrient information) are well supported by the general public and that the introduction of additional obesity-related interventions would also be welcome [20]. However, there is limited data on the beliefs of the general population surrounding living with obesity and its outcomes [21]. Swift et al. aimed to fill this gap by developing the Obesity Beliefs Scale (OBS), a validated measure to assess views about the consequences of living with obesity and healthy weight [22]. OBS can have various applications in research, one of which is exploring the relationship between obesity-related beliefs and weight management behaviour [23–25].

Gaining an insight into public beliefs about the impact of living with obesity and the factors associated with these beliefs may contribute to the design of effective and sensitively designed obesity policies to improve health. This exploratory study aimed to measure public beliefs regarding living with obesity and to identify individuals’ characteristics that are associated with them in a nationally representative sample of adults aged ≥ 18 years drawn from the island of Ireland (RoI and NI). Its objectives were to:

- assess beliefs regarding the consequences of living with obesity and with a healthy weight using the OBS;
- examine the relationship of obesity beliefs with demographics, self-reported weight status, self-perceived health, diet, physical activity and perceived ease to improve them;
- explore any differences between current obesity beliefs and those reported in 2013.

## Methods

A cross-sectional telephone survey was conducted as part of a larger research project exploring public acceptability of policies to address obesity on the island of Ireland. Ethical approval was obtained from University College Dublin Health Research Ethics Committee with a supplementary approval granted by Research Ethics Committee of Faculty of Medicine, Health & Life Sciences, Queen’s University Belfast.

### Participant selection

Participation was open to all adults (over the age of 18) who lived in NI and in RoI and were competent to consent. A random quota sampling approach was utilised with the aim of recruiting a sample of 1000 adults across both countries. Participant selection was based on quotas set for age, gender, socioeconomic status in each jurisdiction.

Telephone recruitment was co-ordinated by a social market research agency who obtained a sample of telephone numbers via an accredited, third-party sample provider to approach potential participants. The sample of telephone numbers (over 10,000) were geo-tagged by geographical area with age, gender and socioeconomic status data to support quota sampling. Both mobile and landline numbers were contacted to reduce data bias, increase the response rate and reach a larger audience.

### Data collection

The survey was piloted on two occasions prior to data collection. Data collection was conducted between October 21 and November 26, 2020 by a team of 15 interviewers who used telephone-based Computer Assisted Personal Interviewing. The interviewers were trained in accordance with ISO20252 standards of social research and 10% of their allocated interviews were monitored by the fieldwork manager to ensure that they were delivered in accordance with agreed procedures.

In the interview preamble, participants were asked to provide consent after receiving information about the anonymity and confidentiality of the survey, duration, risks of participation and their right to withdraw at any time. Names were not collected during the interview. Instead, an identifier which matched each respondent to their data record and phone number was created. Both identifier and phone number were destroyed after 90 days.

The survey lasted for approximately 20 min and captured information on demographics, self-perceived health and lifestyle, and views on living with obesity; informed by previous research. The OBS is a measure of beliefs regarding the consequences of living with obesity and healthy weight for individuals aged 12 years and above [22]. It is a validated, psychometrically sound instrument, written in language suitable for an adult population and has a good level of internal consistency (Cronbach’s alpha = 0.7). It includes 15 items, each one of which is a statement reflecting positive and negative aspects of living with obesity and maintaining a healthy body weight. These items are organised under three subscales on:

1. *Health Beliefs*: relating to the health-related consequences of living with obesity and health benefits of living with a healthy weight (e.g. “*People with obesity need more medical care”*);
2. *Social and Aesthetic Beliefs*: relating to the social and appearance-related costs of living with obesity, as well as relevant benefits of living with a healthy weight (e.g. *“People with obesity would have a better social life if they lost weight”*); and.
3. *Costs*: relating to the costs and difficulties associated with maintaining a healthy weight (e.g. *“Maintaining a healthy bodyweight is expensive”* and *“Maintaining an ideal bodyweight takes a lot of effort”*).

In certain cases, the wording of the original scale items was adjusted to adopt person-first language; for example, the phrase “*an obese person”* was replaced by “*people with obesity”* [26] following significant personal and public involvement in research (PPI) input. Additionally, the seven-point Likert scale response format, originally used by Swift et al. [22], was replaced by a five-point one. This was implemented as the rest of the survey questionnaires followed a 5-point Likert format and incorporating a different number of response options for this one scale may have caused participant confusion and fatigue in this telephone methodology. Previous literature also indicates that scales with lower graduation in response, e.g. 5-point scales, are easier and quicker to use than 7- and 9-point scales and increase response rates [27, 28].

In this survey, item responses were assigned a value ranging from 1 (“Strongly disagree”) to 5 (“Strongly agree”). Exceptions are the items of the Cost Subscale for which scoring is reversed, so that endorsing/agreeing with the costs of healthy weight would result in low scores (1=“Strongly agree”, 5=“Strongly disagree”). Subscale scores were obtained from the summation of item response values and a total OBS score from the summation of the three subscale scores, whereby higher OBS scores indicate greater agreement with the health benefits of a healthy weight and costs of living with obesity.

### Data analysis

Respondents’ Body Mass Index (BMI) was calculated from self-reported height and weight. Living with healthy weight was defined by a BMI value: 18.5–24.9 kg/m^2^; living with overweight by a BMI value: 25-29.9 kg/m^2^; and living with obesity by a BMI value ≥ 30 kg/m^2^. Due to the high non-response rate for height and/or weight (31%), the missing values for BMI were included as a separate value for the variable of weight status (‘non-responders’). Socioeconomic status was determined by the occupation of the highest income earner in participant’s household, according to the RoI Central Statistics Office classification [29], and it was assigned two values for this analysis: ABC1 and C2DE (for professional/managerial and manual/unskilled occupations respectively). Three values were assigned to the educational level based on whether the participants had completed: a compulsory level of education only (i.e., schooling up to 15/16 years of age); a secondary level of education; or a higher education degree.

The data were weighted to represent the population of the island of Ireland in terms of age, gender and socioeconomic status, based on census data [30, 31]. Cases with more than 10% missing data were excluded from the analyses. This practice was not expected to result in a significant loss of statistical power given the large dataset [32]. For the OBS, the score of a single missing item was calculated based upon the average of the remaining items of the corresponding subscale that were scored. A sensitivity analysis was performed to confirm that the results with imputed values were aligned to the results with the missing values.

A Principal Component Analysis (PCA) was conducted to confirm whether the scale items correlated with, and fit under, the three constructs originally described by Swift et al. [22]. Components were extracted based on Eigenvalue to prevent falsely imposing the three components selected by Swift et al. on the present data. Oblimin rotation with Kaiser Normalisation was selected under the assumption that variables are correlated to one another [33]. The strength with which an item significantly loaded on an appropriate component was set to a value ≥ 0.5. The subscales derived from the PCA, along with total OBS, were then assessed for internal consistency assessment using Cronbach’s *α* coefficients. A value of 0.7 was set as the minimum value for an acceptable reliability coefficient [33].

Comparisons between participants with healthy weight and those living with overweight, with obesity and non-responders were performed. For all categorical variables (gender, education, socioeconomic status) chi-squared tests were performed to detect differences between participants in different weight categories. For continuous variables (total OBS score, subscales score, BMI and age), the non-parametric Kruskal-Wallis test was deemed appropriate given the skewed distribution within BMI groups and/or heterogeneity of variance. When the test indicated that differences exist, post hoc analysis using the Mann-Whitney U test was carried out to detect where these differences lie. During the post hoc analysis the Bonferroni correction for repeated comparisons was used setting the level of significance to 0.05/3 = 0.0167 [34].

Multiple linear regression was performed with total OBS score as the outcome variable and all other measured variables (i.e. demographics, self-reported weight status, self-rated health and lifestyle as independent variables). Dummy variables were created for all categorical variables that were included in the regression model: education; BMI; self-perceived health, dietary quality, physical activity; perceived ease to improve diet and physical activity. The reference group for every categorical variable was the one that represented the majority of participants. All analyses were performed using the statistical software package IBM SPSS Statistics 26. *P* values < 0.05 were considered statistically significant.

### Comparison of data over time 2013–2020

Data collected in the current survey were compared to a similar survey that was conducted in 2013 in RoI and captured public perceptions of policies to address obesity [20]. The previous survey obtained ethical approval from the University College Dublin Human Research Ethics Committee and collected data through face-to-face interviews during June and July 2013. Since the previous survey was only conducted in RoI, the 2013 data were compared to the 2020 RoI data only (n = 724). Respondents of the previous survey also completed the OBS and their agreement with the scale items was measured on a 7-point Likert scale (ranging from ‘Strongly Agree’ to ‘Agree’ to ‘Slightly Agree’ etc.). Further details can be found elsewhere [20]. To allow comparisons with 2020 data, the responses for ‘Slightly Agree’ and ‘Agree’ (and similarly for ‘Slightly Disagree’ and ‘Disagree’) were collated to produce a new transformed variable measured on a 5-point scale. This process was performed for all the OBS items. Single missing items were imputed in the same way as described for 2020 data. Internal consistency of the subscales in 2013 data was assessed through Cronbach’s *α* coefficient. Differences between timepoints were explored by running a Mann Whitney U test for the subscale scores (not normally distributed) and independent samples *t*-test for the total OBS score (normally distributed). The Mann Whitney U test was used to compare mean ranks, because the distributions of 2013 and 2020 data did not have the same shape. Multivariable regression was also performed to test differences in subscale scores between timepoints whilst accounting for age, gender, education and BMI score.

## Results

### Principal component analysis

Three cases were removed due to missing data of > 10%. All subscales correlated significantly with one another. Results confirmed that three underlying constructs existed in the data. Items loaded significantly and exclusively onto three components similarly to the original OBS development paper. An exception was, item 14 (i.e. *A person who avoids obesity has a restricted lifestyle*), which did not load significantly on an appropriate factor (loading factor = 0.445). The analysis was repeated without this item and the resulting factor solution accounted for a larger proportion of total variance (58.7%). Therefore, it was decided that further analyses would be carried out without this specific item rendering the total OBS score dependent on 14 items. Additionally, item 15 (i.e. *People with obesity would be treated better if they lost weight*) loaded strongly to the construct of Health Beliefs Subscale, even though in the original paper it corresponds to the Social and Aesthetic Beliefs subscale. This was attributed to the minor change of wording whereby in this survey the statement didn’t include ‘by society’ (this wording amendment was implemented following extensive personal and public involvement (PPI) in the development of the survey). The 14 items included in the OBS along with their corresponding subscales and their loading factors can be seen in Table 1. Cronbach’s α coefficients were: 0.80 for the Health Beliefs Subscale; 0.77 for the Social and Aesthetic Subscale; and 0.78 for the Costs Subscale (Table 2).

**Table 1:** Final principal components analysis pattern matrix

	Factor		
	**1**	**2**	**3**
Health Beliefs Subscale			
People should maintain a healthy weight for optimal health.	**0.822**	0.042	− 0.156
Losing weight would greatly improve the health of people living with obesity.	**0.726**	− 0.118	0.156
A person with a healthy bodyweight can lead a more active life.	**0.686**	0.048	0.136
People with obesity need more medical care.	**0.656**	− 0.013	0.278
People with obesity would be treated better if they lost weight.	**0.528**	0.138	0.263
Costs Subscale			
People have to deny themselves a great deal to avoid obesity.	0.156	**0.741**	− 0.207
Maintaining a healthy bodyweight is expensive.	0.045	**0.737**	0.028
Maintaining a healthy bodyweight is boring.	− 0.212	**0.701**	0.183
Maintaining a healthy bodyweight makes life less fun.	− 0.310	**0.691**	0.350
Maintaining a healthy bodyweight takes a lot of effort.	0.418	**0.684**	− 0.220
Social and Aesthetic Beliefs Subscale			
People with overweight or obesity are considered less attractive.	− 0.013	0.035	**0.802**
People with a healthy bodyweight are taken more seriously.	0.148	0.006	**0.717**
People with obesity are embarrassed by the way they look.	0.259	0.039	**0.613**
People with obesity would have a better social life if they lost weight.	0.427	− 0.039	**0.513**

Final scale items are *N*=14, as one item was eliminated due to insufficient loading onto any of the three constructs

The threshold set for the strength with which an item significantly loaded on a component was ≥ 0.5

A sensitivity analysis showed that the results with imputed values followed a similar pattern to those with the missing values so the analysis with imputed values is presented throughout

**Table 2:** Obesity Beliefs Scale (OBS) total and subscale scores

	Cronbach’s α coefficient	Median	Q1	Q3
Health Beliefs Subscale	0.80	20.0	19.0	23.0
Social and Aesthetic Beliefs Subscale	0.77	15.0	12.0	16.0
Costs Subscale	0.74	14.0	11.0	16.0
Total Obesity Beliefs Scale	0.70	49.0	45.0	52.0

### Participant characteristics and differences based on weight category

Data from 1046 respondents were included in the final analysis. Mean age of respondents was 47 years (range = 18–99). There was approximately an equal split between men and women, as well as between those of higher and lower socioeconomic status. In terms of education, the majority had obtained a second-level or a vocational certification, (i.e., in education until at least age 15/16), and one in five had a university degree. More information on participants’ demographic profile and differences between BMI groups can be found in Table 3. Comparisons based on country of residence (NI and RoI) show that a larger proportion of NI participants had obtained the lowest (compulsory) education level (*p* < 0.001), but also belonged to the higher socioeconomic status (ABC1) (*p* = 0.021), compared to RoI participants (see Additional file - Table 1).

Just under a third of responders (*n* = 327, 31%) did not self-report their weight and height during the telephone survey, preventing the calculation of their BMI (included as a category of ‘non-responders’) (see Additional file - Table 2). Of those, *n* = 6 omitted to provide their height only, *n* = 98 omitted their weight only, whereas *n* = 223 omitted both height and weight data. Omissions of anthropometric measurements were due to respondents either not knowing this information or choosing not to report it; the number of participants falling into each of these two categories is unknown.

Non-responders (for height and weight) differed from those who reported anthropometric measurements in terms of gender (more women did not report versus men), but no other demographic characteristics (see Additional file - Table 3). Almost half of the responders who reported their BMI lived with overweight or obesity (31% and 18% respectively). NI participants reported a significantly higher average BMI score (*p* = 0.001) compared to those living in RoI (Additional file - Table 1).

### Differences in obesity beliefs scale and subscales by weight category

Survey respondents who lived with overweight had significantly lower scores on the Social & Aesthetic Beliefs subscale compared to respondents living with a healthy body weight (*p* = 0.001), showing lower endorsement of the negative impact of living with obesity on one’s social life compared with participants with a healthy body weight. Participants living with obesity had lower scores on the Cost Subscale when compared to respondents living with healthy weight (*p* < 0.001) indicating they endorsed the notion that maintaining a healthy body weight is costly to a greater extent. Participants who did not provide a BMI measurement (non-responders) had lower scores on the Health Beliefs Subscale, on the Social and Aesthetic Subscale and on the Cost Subscale (*p* < 0.001 for all). Differences in scores on the Obesity Beliefs Scale and on the individual subscales can be seen in greater detail in Table 4.

RoI participants scored slightly higher in the Health Beliefs Subscale (*p* = 0.042) than those living in NI; no other cross-country differences were observed in OBS subscales and total score (Additional file - Table 4).

**Table 3:** Demographic and anthropometric characteristics (for overall sample and by weight category)

**Characteristics**	Overall sample	Living with healthy weight ^1,2^ (*n* = 370)	Living with overweight ^1^ (*n* = 223)		Living with obesity ^1^ (*n* = 127)		Non-responders (*n* = 326)	
**Continuous variables**	**Median ** **(Q3-Q1)**	**Median ** **(Q3-Q1)**	**Median (Q3-Q1)**	***P*** ^***3***^	**Median ** **(Q3-Q1)**	***P*** ^***3***^	**Median ** **(Q3-Q1)**	***P*** ^***3***^
age, y (*n* = 1046)	46 (30)	36 (28)	50 (30)	**< 0.001**	58 (21)	**< 0.001**	48 (26)	**< 0.001**
BMI, kg/m^2^ (*n* = 725)	25.02 (6.02)	22.35 (2.92)	26.96 (2.39)	**< 0.001**	34.58 (6.78)	**< 0.001**	–	–
**Categorical variables**	**n (%)**	**n (%)**	**n (%)**		**n (%)**		**n (%)**	***P*** ^***4***^
gender (*n* = 1043)								**< 0.001**
males	493 (47.3)	148 (41.0)	147 (63.7)		76 (58.4)		122 (38.1)	
females	549 (52.7)	213 (59.0)	84 (36.3)		54 (41.6)		199 (61.9)	
education (*n* = 1041)								**< 0.001**
no qualifications or compulsory level	213 (20.5)	59 (16.4)	41 (17.8)		46 (34.5)		68 (21.2)	
secondary/further education (e.g., NVQ)	612 (58.8)	202 (55.9)	141 (61.5)		75 (57.1)		194 (60.8)	
university or higher (UG or PG degree)	216 (20.7)	100 (27.7)	47 (20.6)		11 (8.3)		57 (18.0)	
SES (*n* = 1043)								**< 0.001**
ABC1	450 (43.0)	183 (50.6)	80 (34.5)		43 (32.5)		144 (44.8)	
C2DE	596 (57.0)	178 (49.4)	151 (65.5)		89 (67.5)		177 (55.2)	

^1^BMI range in weight categories: *Living with healthy weight* = 18.5 kg/m^2^ ≤ BMI < 25 kg/m^2^; *Living with overweight* = 25 kg/m^2^ ≤ BMI < 30 kg/m^2^; *Living with obesity =* BMI ≥ 30 kg/m^2^

^2^This group also includes N = 15 participants who had a BMI value between 14.91 and 18.08

^3^*P* values as occurred from *post hoc* Mann-Whitney U test with ‘Living with healthy weight’ as reference group

^4^*P* values as occurred from *χ*^*2*^ test detecting differences between different weight categories

Abbreviations: y = years; NVQ = national vocational qualification; UG = undergraduate; PG = postgraduate; BMI = body mass index; SES = socioeconomic status

**Table 4:** Subscales scores and total Obesity Beliefs Scale scores (for overall sample and by weight category)

Variables	Overall Sample	Living with healthy weight ^1^ (*n* = 370)	Living with overweight ^1^ (*n* = 223)		Living with obesity ^1^ (*N* = 127)		Non-responders ^1^ (*N* = 326)	
	**Median**	**Median**	**Median**	***P*** ^**2**^	**Median**	***P*** ^**2**^	**Median**	***P*** ^**2**^
Health Beliefs Subscale Score (*N* = 1046)	20.00	21.00	21.00	0.028	20.00	0.044	20.00	**< 0.001**
Social And Aesthetic Beliefs Subscale Score (*n* = 1039)	15.00	15.00	14.00	**0.001**	15.00	0.071	14.00	**< 0.001**
Costs Subscale Score (*n* = 1046)	14.00	15.00	14.00	0.033	11.00	**< 0.001**	13.00	**< 0.001**
Total OBS Score ^3^ (*n* = 1039)	48.00	50.00	49.00	**< 0.001**	46.00	**< 0.001**	46.00	**< 0.001**

^1^BMI range in weight categories: *Living with healthy weight* = 18.5 kg/m^2^ ≤ BMI < 25 kg/m^2^; *Living with overweight* = 25 kg/m^2^ ≤ BMI < 30 kg/m^2^; *Living with obesity =* BMI ≥ 30 kg/m^2^

^2^*P* values as occurred from Mann-Whitney U test using ‘Living with healthy weight’ as reference group (significance level set to 0.0167 due to Bonferroni correction for repeated comparisons)

^3^OBS includes 14 items, each one of which can take a value from 1 to 5 rendering a total range of 14 to 70. Higher OBS scores indicate greater agreement with the benefits of living with a healthy weight and negative outcomes of living with obesity

### Association between obesity beliefs scale score and demographics, weight status, self-rated health and wellbeing

As seen in Table 5, the OBS total score was significantly associated with participant self-reported weight status, self-rated overall health, dietary quality and perceived ease of improving diet and physical activity. Participants living with overweight (*p* = 0.001), living with obesity (*p* < 0.001) and those who did not provide a BMI measurement (non-responders) (*p* < 0.001) had lower total OBS scores, indicating less agreement with the negative consequences of living with obesity, compared to participants of a self-reported healthy weight. Additionally, participants who rated their health as ‘very good’ exhibited stronger beliefs in the negative consequences of living with obesity compared with those responding ‘good’ (*p* = 0.013). Rating one’s own diet as ‘quite healthy’ was associated with greater belief in the negative consequences of living with obesity, versus rating one’s diet as ‘not very healthy’ (*p* = 0.004).

When compared with reporting it would be ‘quite easy’ to introduce improvements in one’s diet, reporting it to be quite or very difficult, or stating that no dietary changes are needed were each associated with lower belief in the negative impact of obesity (*p* = 0.012, *p* = 0.001, *p* = 0.028 respectively). People who stated it is difficult (quite or very) to improve their physical activity level and those who stated ‘no changes are needed’ in their physical activity were less likely to agree with adverse outcomes of living with obesity, when compared to those who found it quite easy to introduce those improvements (*p* = 0.035, *p* = 0.001, *p* = 0.012).

**Table 5:** Multivariable regression model with Obesity Beliefs Scale score as outcome variable (N = 1046)

	Unstandardised Coefficient B	95% Confidence Intervals		*P*
**Gender**				
Males	Ref			
Females	-0.365	-1.058	0.327	0.301
**Age**	0.010	-0.011	0.032	0.348
**Socioeconomic status**				
ABC1	Ref			
C2DE	-0.264	-1.051	0.522	0.509
**Education**				
Secondary/further education	Ref			
Compulsory	0.325	-0.576	1.226	0.479
University	0.419	-0.525	1.364	0.384
**Weight status**				
Living with healthy weight	Ref			
Living with overweight	-1.643	-2.598	-0.689	**0.001**
Living with obesity	-2.322	-3.563	-1.081	**0.000**
Non-responders	-3.099	-3.987	-2.211	**0.000**
**Self-perceived health**				
Good	Ref			
Very bad health	-1.870	-5.324	1.585	0.288
Bad health	-1.400	-3.129	0.329	0.112
Fair health	-0.718	-1.724	0.287	0.161
Very good health	1.131	0.240	2.023	**0.013**
**Self-perceived dietary quality**				
Quite healthy diet	Ref			
Very healthy	0.882	-0.246	2.009	0.125
Not very healthy	-1.735	-2.902	-0.567	**0.004**
Not at all healthy	-0.011	-2.221	2.199	0.992
**Self-perceived physical activity**				
Quite active				
Very active	0.508	-0.682	1.697	0.403
Not very active	-0.599	-1.672	0.473	0.273
Not at all active	-0.597	-2.736	1.542	0.584
**Self-perceived ease to improve diet**				
Quite easy	Ref			
Very easy	-0.587	-1.963	0.788	0.402
Quite difficult	-1.554	-2.768	-0.339	**0.012**
Very difficult	-3.442	-5.422	-1.463	**0.001**
No change needed	-1.518	-2.868	-0.168	**0.028**
**Self-perceived ease to improve physical activity**				
Quite easy to improve	Ref			
Very easy	-0.741	-2.146	0.664	0.301
Quite difficult	1.255	0.087	2.424	**0.035**
Very difficult	3.041	1.185	4.898	**0.001**
No change needed	1.788	0.398	3.178	**0.012**

### Change in obesity-related beliefs through time (2013–2020 comparison)

Compared to 2013, respondents in 2020 exhibited stronger beliefs in the health risks associated with living with obesity (*p* < 0.001) (see Table 6). Respondents in 2020 were also more likely to perceive increased costs associated with maintaining a healthy body weight (*p* < 0.001). These differences in the Health Beliefs Subscale and the Cost Subscale scores between the two timepoints persisted when age, gender, education level and self-reported BMI were taken into account. A significant decrease in total OBS scores was observed since 2013 (*p* < 0.001). There were no significant differences in the Social and Aesthetic Subscale scores between time points (*p* = 0.463). Additional file - Table 5 shows the scale properties for both sets of data (2013 and 2020).

**Table 6:** Comparison of Obesity Belief Scale and Subscale scores (2013–2020)

	Mean ranks		*P*
	*2013*	*2020*	
Health Beliefs Subscale	534.51	632.68	**< 0.001** ^**1**^
Social & Aesthetic Beliefs Subscale	584.55	570.04	0.463 ^1^
Costs Subscale	729.95	487.99	**< 0.001** ^**1**^
	**Means**		***P***
Obesity Beliefs Scale	51.10	49.03 ^2^	**< 0.001** ^**3**^

^1^P values as occurred from Mann-Whitney U test

^2^Higher OBS scores indicate greater agreement with the benefits of living with a healthy weight and negative outcomes of living with obesity

^3^P values as occurred from independent samples t-test

## Discussion

This study drew data from a nationally representative sample from the island of Ireland to assess public beliefs regarding living with obesity using the previously validated *Obesity Beliefs Scale (OBS)*. Additionally, it explored the relationship between obesity-related beliefs and self-reported weight status, as well as self-rated health and lifestyle. The present analysis showed that self-reported weight status, self-rated health and dietary quality, along with self-perceived ease to make improvements in one’s own diet and physical activity, are associated with one’s perceptions of the benefits and costs of living with a healthy weight. Results also indicate that differences exist between people living with a healthy weight and those living with excess weight in their perceptions regarding the outcomes of living with obesity. Participants living with overweight and obesity displayed lower endorsement of the benefits of maintaining a healthy weight and the negative outcomes of living with obesity. Specifically, the analyses revealed that people living with overweight supported the social and aesthetic benefits of living with a healthy weight to a lesser extent compared to individuals whose self-reported BMI was within the healthy range. This finding is in agreement with previous studies which have demonstrated that individuals living with obesity may minimise the negative impact of living with excess body weight [35, 36].

Additionally, in the present study individuals with obesity displayed a greater support for the notion that maintaining a healthy body weight comes with higher costs, compared to individuals with a healthy weight. It is possible that this belief may be a result of efforts to manage excess weight in the past, which may have led to weight cycling and regain (which is increasingly recognised as biologically driven via ‘endogenous compensatory mechanisms’ [37]). Indicatively, previous research shows that individuals who perceive their weight status as ‘overweight’ are more likely to have attempted weight loss but also to have gained more weight over time [38]. Furthermore, limited availability of evidence-based treatment options for assisting those with obesity who wish to manage their weight may contribute to ‘unsuccessful’ weight management attempts and hence, to diverging beliefs on the cost associated with maintaining a healthy body weight. One example of such treatment options is publicly-funded bariatric surgery, which is not available in NI via the National Health Service (NHS), and limited in RoI, despite being considered an appropriate obesity treatment for many people with obesity [39, 40]. An alternative explanation for our finding may relate to cognitive dissonance theory [41], according to which individuals living with obesity may attempt to eliminate the inconsistency between their beliefs about obesity and their reality of living with excess weight (which is typically chronic), by emphasising the cost associated with living with a healthy weight. Indeed, when interpreting this finding, a previous cross-sectional analysis of perceptions of obesity should also be noted; its results indicated that individuals living with excess weight perceived obesity to be less controllable compared to healthy-weight individuals [42]. However, this is not always the case, as previous research also highlights that even amongst those living with severe obesity, perceptions around ‘controllability’ and personal responsibility exist, often representing a barrier to accessing support for weight management [43]. The perception that uncontrollable factors may be involved in developing obesity, and hence managing it, is in line with this study’s findings and can partly explain why individuals with obesity may perceive maintaining a healthy weight to be associated with increased effort and cost. Research shows that educating the public with regard to the development of obesity (i.e. it is complex and has many biological, social and psychological drivers, beyond personal control), regardless of their body size, would be of importance [43]. Overall, the underlying factors that influence one’s perceptions of obesity-related risks, along with the barriers faced by those living with obesity remain to be further explored. Further qualitative work could shed light into the more nuanced beliefs regarding the impact of living with obesity.

Findings indicate that people’s beliefs around obesity are associated with their own self-reported weight status, their perceptions of their overall health and diet and how difficult they perceive it would be to improve their lifestyle (diet and exercise). Consistent with findings from a previous cross-sectional analysis [35], this analysis demonstrated that individuals who perceived themselves to be of good health were more likely to endorse the health risks associated with living with obesity. Due to a lack of studies examining how obesity-related beliefs link with self-perceived health and lifestyle, it is difficult to interpret these findings in the context of previous evidence. Our study results however, suggest that people’s beliefs about how easily they can achieve improvements in their diet and exercise are related to their obesity-related views. This finding further emphasises the need for future public health messaging to be framed in a sensitive way to illustrate that the development of obesity is complex and once established, it is a chronic disease [11]. However, emphasising that health gains can be made with health-promoting diet and activity choices for all, regardless of body size, weight loss or weight loss maintenance is important [16].

Current findings point towards a shift in the obesity-related beliefs over time. In 2020, there seems to be greater agreement with the notion that living with obesity has negative health consequences, but also that maintaining a healthy body weight is costly/difficult. The increased support for the negative health outcomes of obesity was expected considering that the current survey was conducted during the COVID-19 pandemic, a time where the increased risk of COVID infection and pathology due to living with excess weight was emphasised [44]. The increased endorsement that maintaining a healthy weight requires effort may also reflect the reality of unhealthy changes in the food environment over time (e.g. the increase in fast food outlets, rise in availability of ultra-processed foods) [45, 46]. It might also be a snapshot of a particular point in time, when due to COVID-19 restrictions (e.g., closure of all indoor physical activity centres) opportunities to be physically active decreased whilst sedentary activities (TV watching) increased [47]. Taken together, these findings provide a reminder that obesity management is an ever-changing landscape and hence, warrant re-investigation of obesity-related beliefs in the future. Present findings also suggest that public information messaging about the health consequences of living with obesity have permeated with the general public, however, it is now time for government and policy action to minimise the costs associated with reducing excess weight and maintaining a healthy body weight [48]. This could be achieved by providing better support and enabling healthier behaviours for all and by investing in treatment options for those with relative higher weight. Findings could also be used to develop attitude/belief modification interventions for specific groups of the population (regardless of their own body size), in order to facilitate future health-promoting behaviours, and decrease the stigmatisation of those with obesity.

## Limitations and strengths

Around a third of people taking part in this survey did not report their height/weight meaning BMI could not be determined. Due to this high volume of missing cases, the non-responders were included in the analysis as a separate group to enable comparisons with people who reported a (self-reported) healthy BMI. Specifically, non-responders scored lower in all OBS subscales compared to healthy weight respondents, revealing lower endorsement of the health, social and aesthetic benefits associated with maintaining a healthy weight. Women had lower response rates to these questions than men, a trend also observed in data of the National Health and Nutrition Examination Survey [49]. Currently, there are not many studies examining factors underpinning non-response rates for self-measurements of height and weight [50, 51]. It is however recognised that identifying oneself with the label of “overweight” or “with obesity” is to identify as being part of a highly stigmatised social group [52]. Additionally, when comparing the proportion of people living with overweight and obesity in this sample to the rates in the RoI and NI population (48% versus 60%) [53, 54], it becomes apparent that respondents may have under-reported their anthropometric characteristics rendering a lower self-reported BMI. Overestimating self-reported height and underestimating weight has been consistently reported in the literature with these trends being more evident among individuals with a BMI over 30 kg/m^2^ [55, 56]. In this study, responders may have felt less comfortable to share their (accurate) anthropometric information with the interviewer over the phone (as opposed to self-reporting them with no one looking/listening). It is also possible that because of the COVID-19 pandemic, which has highlighted issues of excess weight, respondents may have felt hesitant to provide this information, or they may have not known the precise values due to lack of opportunities to weight themselves (e.g., in a healthcare setting during lockdowns).

The OBS provided a useful and flexible tool to assess obesity-related beliefs in our sample. Swift and colleagues considered various methodological aspects and ran rigorous analyses of the subscales’ psychometric properties before establishing the subscales’ construct validity and reliability. Even though the items of the original scale, as developed by Swift et al., were answered on a 7-point Likert scale, in the present survey it was decided that the possible responses should follow a 5-point format. This was a significant modification of the scale, but also crucial due to current circumstances; administering the survey through the phone required the scale to be readily accessible to participants in order to reduce participation burden and maintain good response rates. Certain statements in the scale were also slightly modified to adopt a people-first language, which was considered necessary as part of a wider effort to use appropriate terminology in obesity research [26]. The PCA confirmed that the modified items fit under the original subscales and therefore, it is anticipated that these wording adjustments did not compromise the interpretation of the statements by the survey participants and they do not present a concern for the analysis and interpretation of findings. It is important that researchers also consider their position when researching this area. Best efforts should be made to find terminology in weight-related and stigma research to describe higher-weight bodies and contribute meaningfully to the field, without contributing to the problem [57]. Every effort was made in this research to minimise harm by engaging with a number of PPI representatives and key stakeholders, and adopting person-first language. It has been argued that the very process of labelling (i.e. asking questions about those with obesity versus those of a healthy weight) could exacerbate distinctions in society; however, shedding light on public views about living with obesity as part of this research was considered valuable, to quantify stigmatising views and highlight barriers, in order that they can be proactively addressed [57].

This survey utilised a phone methodology, a reliable approach to measure health behaviours and beliefs [58] with certain advantages over in-person interviews e.g. ability to reach a geographically dispersed sample [59]. Additional strengths of the present study include the large sample, which is representative of populations of RoI and NI, as well as the availability of data to establish time comparisons for RoI.

Finally, the current investigation identified a number of associations between public beliefs about obesity and self-reported weight status, self-rated health, dietary quality, and self-perceived ease to make lifestyle improvements. Even though these associations are noted after adjusting for an array of factors through a multivariable regression, there may be other factors that can explain differences in people’s beliefs about living with obesity. For example, personal experiences of weight-based stigma may be one of them (which can result in higher levels of internalised weight stigma) [7], but given the absence of a validated scale on internalised weight bias in the present study, it was not possible to account for it here.

## Conclusion

Beliefs about living with obesity on the island of Ireland are associated with individuals’ body weight and their own perceptions of their health, diet and perceived ease of adoption of dietary and exercise-related improvements. Beliefs about both the positive and negative implications of living with obesity also appear to have strengthened over time in the RoI. These findings may benefit the design of effective obe;sity policy interventions which seek to combat weight-based discrimination, raise awareness of the health consequences of living with excess weight (alongside highlighting the complex drivers of obesity) and importantly, help to reduce the costs associated with maintaining a healthy body weight via improvements to the food environment.

## Electronic supplementary material

Below is the link to the electronic supplementary material.

Supplementary Material 1

## Data Availability

The datasets used and/or analysed during the current study are available from the corresponding author on reasonable request.

## List of abbreviations

RoI: Republic of Ireland
NI: Northern Ireland
OBS: Obesity Beliefs Scales
BMI: Body Mass Index
PCA: Principal Component Analysis
Y: years
NVQ: national vocational qualification
UG: undergraduate
PG: postgraduate
SES: socioeconomic status
NHS: National Health Service
