# Supplementary material for: Public beliefs about the consequences of living with obesity in the Republic of Ireland and Northern Ireland
Source: BMC Public Health. 2022 Oct 13;22:1910. doi: 10.1186/s12889-022-14280-9 (PMC9559245; doi:10.1186/s12889-022-14280-9)
Supplement: Supplementary file 1 — Supplementary Material 1 [file 12889_2022_14280_MOESM1_ESM.docx]

Table 1. Cross-country comparison of demographic characteristics

|  | Northern Ireland  (n=322) | Republic of Ireland  (n=724) |  |
| --- | --- | --- | --- |
|  | Mean (SD)/n (%) | Mean (SD)/n (%) | P |
| Gender |  |  | 0.893^1^ |
| Males / Females | 47.8% / 52.2% | 47.1% / 52.9% |  |
| Age (years) | 44.4 (16.98) | 47.4 (17.52) | 0.044^2^ |
| Education level |  |  | < 0.001^1^ |
| Compulsory level /  Secondary - further education / University degree | 30.0% / 49.9% / 20.1% | 16.2% / 62.8% / 21.0% |  |
| Social class |  |  | 0.021^1^ |
| ABC1 / C2DE | 48.3% / 51.7% | 40.6% / 59.4% |  |
| BMI (Kg/m^2^) | 27.4 (7.07) | 25.61 (5.22) | 0.001^2^ |

^1^ P values as occurred from χ^2^ test; ^2^ P values as occurred from Mann-Whitney U test

Table 2. Body Mass Index (BMI) categories across the sample (including proportion of non-responders)

|  | N (%) |
| --- | --- |
| Living with healthy weight | 370 (35.3%) |
| Living with overweight | 223 (21.3%) |
| Living with obesity | 127 (12.2%) |
| Doesn’t know / refused to say | 326 (31.2%) |

Table 3. Demographic comparison between BMI responders and non-responders

|  | Responders | Non-Responders | P |
| --- | --- | --- | --- |
| Gender |  |  | **< 0.001** ^1^ |
| Males / Females | 35.6% / 33.7% | 11.7% / 19.1% |  |
| Age (years) | 45.9 | 47.9 | 0.078 ^2^ |
| Education level |  |  | 0.337 ^1^ |
| Compulsory level /  Secondary - further education / University degree | 14.0% / 40.2% / 15.2% | 6.5% / 18.6% / 5.5% |  |
| Social class |  |  | 0.456 ^1^ |
| ABC1 / C2DE | 29.3% / 40.1% | 13.8% / 16.9% |  |

^1^ P values as they occurred from χ^2^-square test; ^2^ P value as it occurred from Student’s t-test

Table 4. Cross-country comparison of Obesity Beliefs Scale and Subscales

|  | Northern Ireland  (n=322) | Republic of Ireland  (n=724) |  |
| --- | --- | --- | --- |
|  | Mean Rank | Mean Rank | P ^3^ |
| Health Beliefs subscale score ^1^ | 495.58 | 536.20 | **0.042** |
| Social and Aesthetic Beliefs subscale score ^1^ | 532.27 | 515.12 | 0.390 |
| Costs subscale score ^1^ | 497.27 | 535.43 | 0.057 |
|  | Mean (SD) | Mean (SD) | P ^4^ |
| Total OBS score ^2^ | 48.24 (5.66) | 48.76 (6.02) | 0.188 |

^1^ Measured on a continuous scale with normal distribution and similar variance within RoI and NI groups

^2^ Measured on a continuous skewed scale within RoI and NI groups

^3^ P values as they occurred from Mann-Whitney U test and significance level was set to 0.05

^4^ P value as it occurred from Student’s t-test and significance level was set to 0.05

Table 5. Cronbach’s alpha coefficients for OBS and subscales for both sets of data (2013-2020)

|  |  | Cronbach’s α coefficient | | | | |
| --- | --- | --- | --- | --- | --- | --- |
|  | | |  | 2013 | | 2020 |
| Health Beliefs Subscale | | |  | 0.69 | | 0.80 |
| Social & Aesthetic Beliefs Subscale | | |  | 0.64 | | 0.77 |
| Costs Subscale | | |  | 0.67 | | 0.74 |
| Obesity Beliefs Scale | | |  | | 0.74 | 0.70 |
